# Assessing the Outcomes of Patients with Severe SARS-CoV-2 Infection after Therapeutic Plasma Exchange by Number of TPE Sessions

**DOI:** 10.3390/jcm12051743

**Published:** 2023-02-22

**Authors:** Tamara Mirela Porosnicu, Dorel Sandesc, Daniel Jipa, Ciprian Gindac, Cristian Oancea, Felix Bratosin, Roxana Manuela Fericean, Shiva Charana Kodimala, Ciprian Nicolae Pilut, Laura Alexandra Nussbaum, Ioan Ovidiu Sirbu

**Affiliations:** 1 Doctoral School, “Victor Babes” University of Medicine and Pharmacy Timisoara, 300041 Timisoara, Romania; 2 Intensive Care Unit, “Victor Babes” Clinical Hospital for Infectious Diseases and Pneumology, 300041 Timisoara, Romania; 3 Department of Anesthesia and Intensive Care, “Victor Babes” University of Medicine and Pharmacy Timisoara, 300041 Timisoara, Romania; 4 Intensive Care Unit, “Pius Brinzeu” Emergency Clinical Hospital, 300041 Timisoara, Romania; 5 Center for Research and Innovation in Precision Medicine of Respiratory Disease, “Victor Babes” University of Medicine and Pharmacy Timisoara, 300041 Timisoara, Romania; 6 Department XIII, Discipline of Infectious Disease, “Victor Babes” University of Medicine and Pharmacy Timisoara, 300041 Timisoara, Romania; 7 MediCiti Institute of Medical Sciences, NTR University of Health Sciences, Hyderabad 501401, India; 8 Department of Microbiology, “Victor Babes” University of Medicine and Pharmacy Timisoara, 300041 Timisoara, Romania; 9 Department of Neurosciences, “Victor Babes” University of Medicine and Pharmacy Timisoara, 300041 Timisoara, Romania; 10 Center for Complex Network Sciences, “Victor Babes” University of Medicine and Pharmacy Timisoara, 300041 Timisoara, Romania

**Keywords:** therapeutic plasma exchange, plasmapheresis, SARS-CoV-2, COVID-19

## Abstract

The high mortality risk in severe SARS-CoV-2 infections is tightly correlated to the extreme elevation of inflammatory markers. This acute accumulation of inflammatory proteins can be cleared using plasma exchange (TPE), commonly known as plasmapheresis, although the available data on performing TPE in COVID-19 patients is limited regarding the optimal treatment protocol. The purpose for this study was to examine the efficacy and outcomes of TPE based on different treatment methods. A thorough database search was performed to identify patients from the Intensive Care Unit (ICU) of the Clinical Hospital of Infectious Diseases and Pneumology between March 2020 and March 2022 with severe COVID-19 that underwent at least one session of TPE. A total of 65 patients satisfied the inclusion criteria and were eligible for TPE as a last resort therapy. Of these, 41 patients received 1 TPE session, 13 received 2 TPE sessions, and the remaining 11 received more than 2 TPE sessions. It was observed that IL-6, CRP, and ESR decreased significantly after all sessions were performed in all three groups, with the highest decrease of IL-6 in those who received >2 TPE sessions (from 305.5 pg/mL to 156.0 pg/mL). Interestingly, there was a significant increase in leucocyte levels after TPE, but there was no significant difference in MAP changes, SOFA score, APACHE 2 score, or the PaO2/FiO2 ratio. The ROX index was significantly higher among the patients who underwent more than two TPE sessions, with an average of 11.4, compared to 6.5 in group 1 and 7.4 in group 2, which increased significantly after TPE. Nevertheless, the mortality rate was very high (72.3%), and the Kaplan–Meier analysis identified no significant difference in survival according to the number of TPE sessions. TPE can be used as last resort salvage therapy that can be regarded as an alternative treatment method when the standard management of these patients fails. It significantly decreases the inflammatory status measured via IL-6, CRP, and WBC, as well as demonstrating an improvement of the clinical status measured via PaO2/FiO2, and duration of hospitalization. However, the survival rate does not seem to change with the number of TPE sessions. Based on the survival analysis, one session of TPE as last resort treatment in patients with severe COVID-19 proved to have the same effect as repeated TPE sessions of 2 or more.

## 1. Introduction

The novel coronavirus SARS-CoV-2 posed a significant challenge to the whole global health system owing to the high number of patients requiring hospitalization in a short period of time, resulting in a worldwide health catastrophe [1,2,3]. During the past three years of the COVID-19 pandemic, a tremendous amount of scientific and clinical work has been done to find solutions for the prevention and treatment of SARS-CoV-2 infections. However, there are still many gaps in our knowledge, and an effective treatment is yet to be found, particularly when it comes to those affected by a severe form of COVID-19 [4,5,6].

Among the hypothesized treatment methods, there was an attempt to reduce the severe inflammation that occurs in severe COVID-19, triggering acute respiratory distress syndrome (ARDS) [7,8,9]. Multiple studies have identified a rise in lactate dehydrogenase (LDH), ferritin, fibrinogen, IL-6, C-reactive protein, and D-dimers and a decrease in lymphocytes as biological inflammatory indicators [10,11,12]. Many of the abnormalities seen in hospitalized patients are caused by a cytokine storm, which is defined by an excessive host reaction to the virus and shares similarities with bacterial septic shock and catastrophic negative fulminant evolution. Alternatively, ARDS and cytokine storm syndrome, leading to multisystem organ failure, are the primary causes of mortality in patients with COVID-19 infection [13,14].

Despite the fact that vaccines are currently offered in most of regions worldwide, there are constraints in some regions because of problems with vaccine acceptance [15,16,17,18], where the proportion of fully vaccinated population is low, and there have been new outbreaks in 2023, even in regions where the majority of the population received two or three doses of COVID-19 vaccine [19,20]. Thus, clinicians continue to encounter severe COVID-19 patients that need increasingly sophisticated medications and distinct management strategies.

Depending on the characteristics of the population, the mortality rate in severe cases of COVID-19 remains substantial [21,22]. Despite the fact that various potentially valuable treatment techniques have been researched and attempted, only a few of them have been shown to be effective, and only under certain conditions. For instance, TPE, also known as plasmapheresis, is a medical technique in which a patient’s plasma is separated from pathologic components of their blood then replaced with either an albumin solution or fresh frozen plasma (FFP) [23,24]. The basic goal of TPE in a variety of conditions is to eliminate pathological elements from the patients’ blood, such as antibodies and inflammatory proteins, toxic substances like drugs and toxins, and for the correction of blood coagulation disorders. All these properties give TPE a wide range of uses as first line treatment for conditions like glomerulonephritis, myasthenia gravis, Guillain-Barre syndrome, Wilson disease, thrombotic thrombocytopenic purpura, and many others [25].

Since a cytokine storm-mediated immune response triggers many complications and organ damage, it was hypothesized that eliminating harmful antibodies and cytokines might lower the severity of the disease. In light of these factors, TPE has been suggested as a viable therapeutic option for severe SARS-CoV-2 infections, although being reserved for those cases where other options failed. Therefore, the objective of this scientific project was to observe the role of plasma exchange therapy in lowering the level of inflammation in critically ill COVID-19 patients and whether it prevents severe complications and to find the optimal treatment protocol to improve patients’ survival according to the number of plasmapheresis sessions.

## 2. Materials and Methods

### 2.1. Study Design and Ethics

Between March 2020 and March 2022, the research gathered data from the Clinical Hospital for Infectious Diseases and Pneumology in Timisoara, which accepted SARS-CoV-2-infected patients from western Romania. During this time period, several patients with severe forms of COVID-19 who required intubation and mechanical breathing were hospitalized in the intensive care unit (ICU). Patients admitted to the ICU with ARDS within this time frame were evaluated for inclusion in this research. It was found that exchange therapy was performed in 65 adult patients with significant respiratory impairment and no response to other treatments.

The study took place in the intensive care unit of the Clinical Hospital for Infectious Diseases and Pneumology in Timisoara, which is affiliated with the Victor Babes University of Medicine and Pharmacy Timisoara. Signed informed permission was acquired from all participants or their legal guardians. The hospital’s Ethical Committee authorized the research for data collection and outcomes publication on 24 December 2021, with permission number 13037. 

### 2.2. Selection Criteria and Study Variables

Patients were identified from the hospital database and their associated paper records with all treatments, procedures, and laboratory analyses were registered. The inclusion criteria comprised the following particularities: (1) patients required to be at least 18 years old; (2) a diagnosis of COVID-19 established via a positive PCR test at the moment of admission [26]; (3) patients must have moderate or severe ARDS and/or cytokine storm development, identified via an excessive rise in particular markers for systemic inflammation; (4) patients must have had their clinical and biological data measured before and one day after plasmapheresis sessions; (5) written informed consent must have been identified in the personal paper records of the patients. These patients exhibited clinical respiratory distress necessitating intensive care unit hospitalization and required additional oxygen supplementation of more than 30 L/min. Other parameters that suggest ICU admission include: SpO2 levels below 90%, respiratory rate greater than 30 breaths/min with signs of respiratory failure, and PaO2/FiO2 200 of mmHg (moderate ARDS = PaO2/FiO2 of 200 mmHg, or severe ARDS = PaO2/FiO2 of 100 mmHg).

The current study analyzed the following variables: body mass index, age, age range, gender (male, female), the duration from COVID-19 diagnosis and TPE, mechanical ventilation (invasive, non-invasive), number of comorbidities, comorbidities (diabetes mellitus, hypertension, obesity, COPD/asthma, others), the laboratory profile (IL-6, ferritin, D-dimers, CRP, LDH, procalcitonin, fibrinogen, ESR, leucocytes, lymphocytes, BUN, creatinine, pH, lactate), mean arterial pressure (MAP), body temperature, SOFA score, APACHE2 score, PaO2/FiO2 ratio, and mortality rate.

### 2.3. Definitions and TPE Procedure

To assess the effect of organ failure after the TPE session, liver enzymes, blood urea nitrogen (BUN), creatinine, pH, lactate, SOFA score [27], and APACHE II score [28] were analyzed. Patients’ oxygenation status was evaluated using PaO2/FiO2 and respiratory rate, ROX index score [29], HACOR score [30], and the oxygenation index (OI). The TPE sessions were conducted using either a Prismaflex (Baxter International, Deerfield, IL, USA) device with a TPE 2000 plasma-filter or a HF404 (Infomed) machine with a Granopen 60 plasma-filter. As replacement fluid, between 1 and 1.3 times the plasma volume units of the patient was exchanged in doses of 5% human albumin solution or FFP. As an anticoagulant, fractionated heparin and/or citrate were utilized. The patients who received TPE did not get plasma from convalescent patients, but only clear plasma for transfusion. In the hospital laboratory, biochemical parameters were determined using a COBAS INTEGRA 400 plus instrument (Roche Diagnostics), and the reverse transcription polymerase chain reaction (RT-PCR) for SARS-CoV-2 testing was done using RT-PCR equipment (Bio-Rad, Hercules, CA, USA). 

### 2.4. Statistical Analysis

The statistical analysis was performed using GraphPad Prism version 6.0 for Microsoft Windows (GraphPad Software La Jolla, CA, USA). Due to the lengthy and costly procedure of TPE, only 65 patients benefitted from TPE, therefore, the sample size and statistical power calculation were considered irrelevant. Data normality was tested with the Kolmogorov-Smirnov test. Normally distributed data were presented according to the mean value, as representative for central tendency, and the standard deviation as a measure of dispersion. The difference between the means of the three comparison groups was tested with the ANOVA test. Non-normally distributed data were described using the median and interquartile range (IQR) and compared with the Kruskal–Wallis test. Proportions were presented as *n* (%) and compared with the Chi-square test or the Fisher’s exact test, if the frequency assumption was not met for the Chi-square test. The test significance was described using a “*p*-value”, where a “*p*-value” lower than 0.05 was considered statistically significant.

## 3. Results 

### Patients’ Background Characteristics

Among the 65 participants, there were 41 patients who received only one session of TPE, thirteen who received 2 sessions, and the other eleven patients received more than two TPE sessions. Table 1 presents a comparison of baseline characteristics of patients with severe COVID-19 according to the number of TPE sessions. There were no significant differences between the three study groups regarding their body mass index, age, gender, mechanical ventilation necessities, and existing comorbidities. The average age of patients with severe COVID-19 who received TPE was around 50 years, with an average of 54.4 years in group 1 (1 TPE session), 50.8 years in group 2 (2 TPE sessions), and 47.8 years in group 3 (>2 TPE sessions). It is important to mention that in all three groups, the BMI was higher than 32, which corresponds to class I obesity. The gender distribution of these patients indicated a high proportion of men, with an average of 70.4% across the entire cohort. Being admitted to the ICU with severe COVID-19, the majority of patients necessitated invasive ventilation, without a significant difference between groups (58.5% in group 1 vs. 76.9% in group 2 vs. 63.6% in group 3). Similarly, there were no significant differences with regards the number of comorbidities, where the most common was hypertension.

Regarding the blood analysis measured at admission in the ICU, the laboratory findings of patients with severe COVID-19 before TPE are presented in Table 2. It was observed that IL-6 levels were significantly more elevated among patients who underwent more than three sessions of TPE, with a median value of 305.5 pg/mL, compared to 108.1 in group 1 and 37.0 in group 2 (*p*-value < 0.001). In addition, CRP levels were more elevated in group 3, compared to the other two (111.0 mg/L vs. 88.0 in group 1 and 32.5 in group 2, *p*-value = 0.001). Although CRP and IL-6 were higher in group 3 patients, the median value of leucocytes was significantly higher in group 2 patients who underwent 2 TPE sessions (17.0 vs. 12.7 in group 1 vs. 11.9 in group 3, *p*-value < 0.001). Furthermore, BUN was also the highest in group 2, with a median value of 71.5 mg/dL, compared to 54.0 mg/dL in group 1 and respectively 56.2 in group 3 (*p*-value = 0.036). 

One day after the first session of TPE was performed, the same blood parameters were measured to compare the changes that have occurred, as presented in Table 3. IL-6 levels remained significantly different between the three study groups, but they also decreased significantly after the first TPE. However, the highest decrease in IL-6 levels was measured in patients with more than two sessions of plasmapheresis, decreased from 305.5 pg/mL to 156.0 pg/mL (*p*-value < 0.001). LDH also decreased significantly in all three groups, with approximately 100 units in each group of patients. Regarding the CRP levels, the difference between groups remained significantly high, but there was no significant difference overall between the values measured before and after TPE. ESR decreased significantly after the TPE, although they remained the highest in group 3 of patients who received more than 2 sessions of TPE. Lastly, there was a significant increase in leucocyte levels after the TPE was performed; in group 1 they increased from 12.7 to 15.2, in group 2 from 17.0 to 18.5, while in group 3 they increased from 11.9 to 14.2 ×10^3^/uL (*p*-value = 0.003). 

**Figure 1:**
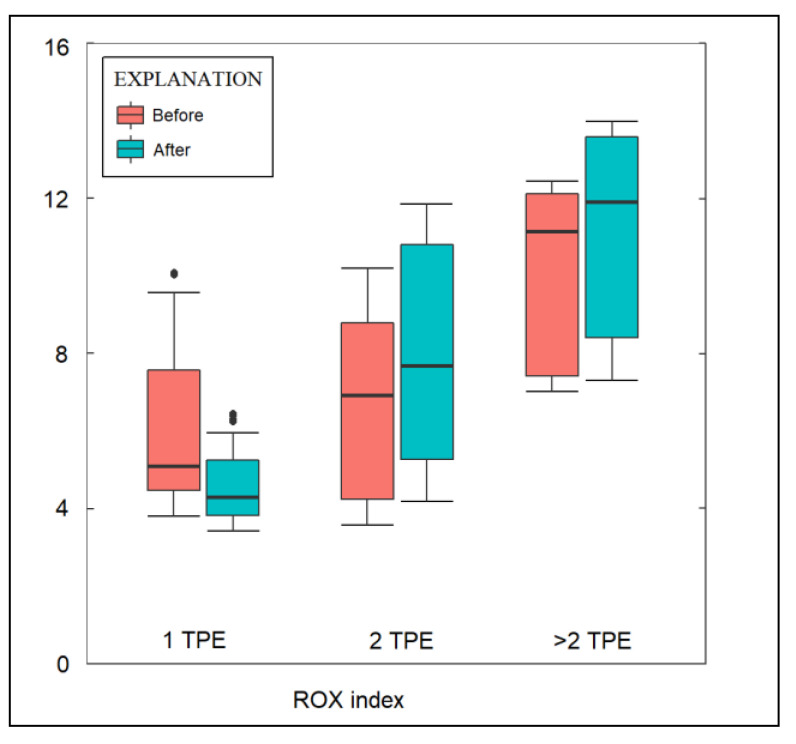
The influence of TPE on the ROX index by number of TPE sessions.

**Figure 2:**
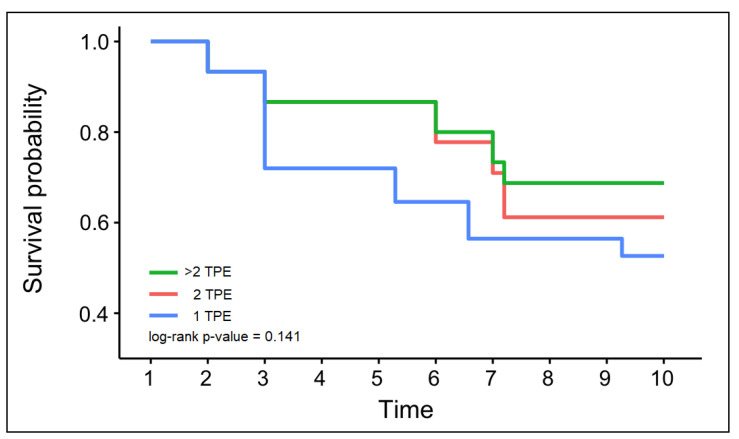
Kaplan–Meier survival analysis by number of TPE sessions. Time was measured in days.

Table 4 presents the outcomes of patients with severe COVID-19 according to the number of TPE sessions. It was observed that the mean arterial pressure did not differ significantly between the three study groups before TPE, but the difference became statistically significant after TPE, with an average of 75.0 mmHg in group 1, compared to 88.7 mmHg in group 2, and 80.6 mmHg in those who received more than 2 TPE sessions (*p*-value = 0.023). There was no significant difference in MAP change after TPE compared to the same groups before TPE. Among the studied parameters, neither the SOFA score, nor the APACHE 2 score, the oxygenation index, or the PaO2/FiO2 ratio changed significantly the following day after undergoing TPE. Similarly, there was no difference regarding the SOFA score, the APACHE 2 score, the oxygenation index, or the PaO2/FiO2 ratio among the studied groups before or after undergoing TPE. However, it was observed that the ROX index was significantly higher among the patients who underwent more than two TPE sessions, with an average of 11.4, compared to 6.5 in group 1 and 7.4 in group 2 (*p*-value = 0.007). Furthermore, the ROX score increased after TPE, but without statistical significance, and it remained the highest in group 3, with an average of 13.1, as presented in Figure 1. Regarding the proportion of intubated patients, there were 18 (43.9%) in group 1, 5 (38.5%) in group 2, and 5 (45.5%) in group 3. The mortality rate was even higher, since only 18 patients survived. The mortality rate at five days and the overall mortality did not show significant differences between the study groups with one or multiple TPE sessions. A Kaplan–Meier analysis was performed to estimate the probability of survival based on the number of TPE sessions performed. As presented in Figure 2, there was no significant difference between the three study groups (log-rank *p*-value = 0.141).

## 4. Discussion

### 4.1. Literature Findings 

Plasma exchange is thought to enhance organ function by eliminating inflammatory and antifibrinolytic mediators, maintaining endothelial membranes, and replacing anticoagulant proteins to restore hemostasis. In the early stages of sepsis, the elimination of these chemicals may be beneficial [31,32]. Given the enormous number of severe/critical patients with SARS-CoV-2 infection and the absence of a particular therapy, numerous therapeutic procedures have been seen as urgent life-saving interventions. One of these treatments is plasma exchange therapy. This approach eliminates endogenous and exogenous inducers of the systemic inflammatory response, pro-inflammatory mediators, cytokines, and reactive oxygen species [33]. Other researchers examined 87 severely sick COVID-19 patients in a study where 43 received plasmapheresis and standard treatment, while 44 received only standard treatment. The authors reported that patients who received TPE exhibited large and persistent increases in lymphocyte count and reductions in CRP, LDH, ferritin, D-dimers, and Il-6. This research reveals that TPE decreases inflammatory markers and improves oxygenation and clinical condition in COVID-19 patients with life-threatening manifestations. However, it has no substantial effect on 35-day mortality [34]. Moreover, in a trial including 31 COVID-19 patients, TPE was beneficial for 11 patients who survived. After plasmapheresis was performed, there was a substantial drop in inflammatory markers and an increase in lymphocyte count. In terms of extubation and death rates, patients receiving TPE showed a better clinical course compared to those in the control group [35].

In separate research including just seven patients, it was shown that all of them had elevated inflammatory markers before TPE and afterwards had favorable clinical and biological outcomes. According to the authors, the better outcome was attributable to the timely commencement of TPE on the first day of the cytokine storm, before intubation and mechanical breathing were required [36]. In a pilot cohort trial including only 10 patients with severe SARS-CoV-2 infections, virtually every patient had five TPE sessions, and none of the patients had previously received corticosteroids, IL-6 inhibitors, Remdesivir, or convalescent plasma. It was found that 14 days after initiating TPE, substantial decreases in CRP and IL-6 were noted, in addition to an improvement in respiratory status [37].

Plasma exchange can modify pathogenic inflammatory drivers in seriously ill COVID-19 patients, which, instead of protecting the organism, produce an exaggerated response to the virus. Depending on their molecular weight, this may decrease D-dimer levels, indicating that the patient’s condition has been artificially reduced rather than cured. Moreover, our findings are consistent with those of other studies in which the authors demonstrated a beneficial effect of plasma exchange by demonstrating decreased fatality rates in patients with D-dimers greater than or equal to 2 mg/L on plasma exchange therapy compared to patients with D-dimers >2 mg/L who did not receive plasma exchange [38]. Despite the severity of the disease in this sample, data further supports the idea that PE improves survival by indicating a 30-day mortality rate of 20% in patients treated with PE as opposed to 50% in patients getting conventional treatment.

Relative to the reference group, plasmapheresis improved the mortality of patients with severe COVID-19 in our research. Various case series have regularly indicated a decrease in 28-day fatality related with TPE, ranging from 10% to almost 30% in patients with severe COVID-19, ARDS, and cytokine release syndrome. In one major trial, patients admitted with SARS-CoV-2 infection and cytokine storm syndrome were randomly assigned to undergo two sessions of TPE or conventional treatment, although it is unclear why it was considered that two sessions of TPE are sufficient [39]. In comparison, in our study, patients received different treatment schemes, most of them undergoing just one plasmapheresis session. The primary outcome in that trial was all-cause death at 60 days; secondary outcomes were mechanical ventilation need, SOFA score alteration, decrease of pro-inflammatory biomarkers, and hospitalization duration. Forty patients undergoing conventional treatment were evaluated compared to 20 individuals undergoing TPE. It was observed that TPE decreased fatality at 60 days by 30%, and this impact was independent of demographic characteristics and pharmacological therapy used. Plasmapheresis lowered the SOFA score and pro-inflammatory markers and raised the lymphocyte count, with a tendency toward a reduction in affected lung capacity, but had no impact on the SatO2/FiO2 indicator or the need for mechanical ventilation [39]. Similarly, our study also did not observe important changes in the PaO2/FiO2 after TPE, but the scores improved, the inflammatory markers decreased, and the lymphocyte count increased, although with no significant difference. In patients with COVID-19, TPE treatment produced considerable advantages of pro-inflammatory clearance and decrease of 60-day mortality without major adverse effects.

In another trial, patients with severe COVID-19 underwent 5 sessions of TPE, compared with our study where the majority of patients received only 1 TPE session. In the respective study, there were no deaths recorded (0 vs. 35% in patients undergoing regular treatment for COVID-19) and no side events were reported [35]. This may be owing to the fact that just one patient in the TPE group had serious pneumonia, while fifty percent of patients in the control group did. It is plausible that the influence of TPE is influenced by the removal of pro-inflammatory markers and harmful biological compounds, representing a potential therapeutic option for patients with a hyperinflammatory state that triggers multiple severe complications caused by SARS-CoV-2 infection [40,41]. In our investigation, TPE was efficient at removing pro-inflammatory cytokines and reducing mortality; nevertheless, it did not avoid the need for mechanical ventilation, indicating that pulmonary insufficiency may be caused by other illnesses. 

Nevertheless, the principle of removing inflammatory markers during the acute phase of severe COVID-19 infection still continues to be a plausible treatment method, considering the effects might vary from case to case individually. IL-1 has a crucial role in producing the cytokine storm that emerges in severe COVID-19, according to many studies [42]. IL-1 induces bronchial and alveolar inflammation in individuals with damaged pulmonary tissue. In addition, it may increase the production of acute phase reactants due to hepatocytes. Several investigations have shown that IL-1 regulates the production of IL-6, one of the primary unfavorable prognostic markers in COVID-19 [42]. Patients infected with SARS-CoV-2 may have rapid lung damage and cytokine storm due to uncontrolled IL-1 beta production. In light of the above, inhibiting or removing IL-1 and IL-6 may be of great use in the treatment of cytokine storm syndrome.

Besides the cytokine storm that is evident in severe COVID-19, there have been many reports regarding the coagulation disorders seen in these patients. One study aimed to evaluate the efficacy of TPE in correcting coagulation disorders in 60 critically ill COVID-19 patients [34]. The patients underwent TPE treatment, and their coagulation parameters were monitored before and after the procedure. The results showed that TPE was effective in correcting coagulation disorders in critically ill COVID-19 patients. The TPE treatment reduced the levels of D-dimer, a marker of coagulation activation, and improved the prothrombin time and activated partial thromboplastin time, which are measures of coagulation function. The authors concluded that TPE is an effective treatment option for correcting coagulation disorders in critically ill COVID-19 patients. The procedure is safe and well-tolerated, and it can improve the outcomes of patients with severe COVID-19 and coagulation disorders. 

Another study evaluated 20 critically ill COVID-19 patients with severe respiratory failure. The patients underwent TPE treatment, and their cytokine levels and respiratory function were monitored before and after the procedure [43]. The results showed that TPE was associated with a significant reduction in the levels of cytokines in the blood and an improvement in respiratory function measured by a reduction in the required oxygen delivery support in patients of the same age range. Alharty et al. included 50 patients with severe COVID-19 and respiratory failure, and their respiratory function was monitored before and after the TPE procedure [44]. The results showed that TPE was associated with a significant reduction in the levels of cytokines in the blood and an improvement in respiratory function. The authors concluded that TPE is an effective treatment option for improving respiratory function in severe COVID-19 patients.

The reduction of inflammatory cytokines and improvement of serum markers in patients with severe COVID-19 was described in a recent systematic review that included 267 patients [45]. IL-6 readings reduced from a high median of 1233 ug/L to 290 ug/L, whereas CRP values decreased from a high median of 344 mg/L to 25 mg/L. However, there was no association with the number of TPE sessions, which was one of our research objectives. The researchers stated that, despite differences across trials, a general tendency was seen for lower interleukin-6, C-reactive protein, ferritin, d-dimer, and fibrinogen levels and increased lymphocyte counts after TPE, suggesting the immunomodulatory action of this therapy. In addition, TPE was related with improved clinical outcomes in severely sick COVID-19 patients.

Even from the beginning of the new coronavirus pandemic, interleukin-6 (IL-6) has been a prognostic biomarker with significant clinical value that has distinguished between mild, moderate, and severe forms of the disease, raising an important question: if it could be controlled, could the SARS-CoV-2 infection evolution be halted? In several studies, attempts have been made to manage IL-6 by maintaining it within normal levels using a variety of medical techniques. One of the techniques was plasma exchange therapy, which dramatically decreased IL-6 levels [46]. In research involving over 300 patients done in the United States, IL-6 was shown to be the most accurate biomarker for predicting illness severity in individuals infected with the novel coronavirus. IL-6 is associated with length of hospitalization, illness severity, and prognosis. The author noted that the test is applicable for triage of infected patients [47].

### 4.2. Study Limitations and Future Perspectives

Among the limitations of the current study is the retrospective design itself. Since it was not organized as a clinical trial, there are many factors that could not be controlled. Ideally patients would be selected with similar characteristics that will not alter the effect of TPE. In our study, heterogeneity was high in all three groups, however, the laboratory findings before undergoing TPE indicated that there were not too many differences in the inflammatory status of the patients in each group. Another limitation of this study is the outcome measure, since only the short-term outcomes were measured. TPE treatments may assist in reducing the virus load, but combining TPE with passive antibody transfer from convalescents could be a worthwhile avenue for further exploration. The benefits and potential drawbacks of this approach would need to be thoroughly evaluated before making any conclusions. Further research is needed to fully understand the role of antibodies in the context of SARS-CoV-2 infection and to accurately evaluate their potential impact on the outcome of TPE treatments.

## 5. Conclusions

Almost three years have passed since the onset of the pandemic, and the complex mechanism of the disease is not fully known, and the race to find an efficient treatment is still not over. From the available data, we found that TPE might be considered a complementary alternative therapy for COVID-19 patients with severe illness. Even after one TPE session it was observed that there was a substantial decrease in inflammatory mediators, improvement in coagulation function, and improvement in clinical status, in a non-inferior way compared to patients who underwent two or more sessions of plasmapheresis. The respiratory function was however significantly improved after two or more TPE sessions compared to only one session, but overall, the survival was not significantly influenced. However, the available literature and their outcomes are very variable, with the majority of them failing to indicate an improvement compared to a control group. Nevertheless, this method has not yet been statistically verified in trials with a larger number of patients in order to be considered reliable in terms of a proper study protocol and a significant improvement in patients’ survival.

## Figures and Tables

**Table 1:** Comparison of baseline characteristics of patients with severe COVID-19 by number of TPE sessions.

Variables	1 TPE (*n* = 41)	2 TPE (*n* = 13)	>2 TPE (*n* = 11)	*p*-Value
BMI (mean ± SD)	33.7 ± 6.2	32.4 ± 5.9	33.1 ± 6.0	0.793 *
Age (mean ± SD)	54.4 ± 12.5	50.8 ± 14.6	47.8 ± 9.5	0.262 *
Age range, years	28–80	21–72	37–69	
Gender				0.775
Male	29 (70.7%)	10 (76.9%)	7 (63.6%)	
Female	12 (29.3%)	3 (23.1%)	4 (36.4%)	
Days from COVID-19 diagnosis and TPE (mean ± SD)	12.7 ± 8.9	13.7 ± 6.0	11.6 ± 5.8	0.814 *
Mechanical ventilation				0.488
Invasive	24 (58.5%)	10 (76.9%)	7 (63.6%)	
Non-invasive	17 (41.5%)	3 (23.1%)	3 (36.4%)	
Number of comorbidities (*n*,%)				
Diabetes mellitus	6 (14.6%)	2 (15.4%)	2 (18.2%)	0.958
Hypertension	20 (48.8%)	7 (53.8%)	6 (54.5%)	0.915
Obesity	17 (41.5%)	6 (46.2%)	4 (36.4%)	0.888
COPD/asthma	4 (9.8%)	2 (15.4%)	0 (0.0%)	0.423

Data reported as *n* (%) and calculated using Chi-square test and Fisher’s exact unless specified differently; SD—standard deviation; TPE—therapeutic plasma exchange; BMI—body mass index (kg/m^2^); COPD—chronic obstructive lung disease; * ANOVA test.

**Table 2:** Laboratory findings of patients with severe COVID-19 before TPE.

Variables—Median (IQR)	1 TPE (*n* = 41)	2 TPE (*n* = 13)	>2 TPE (*n* = 11)	*p*-Value
IL-6, pg/mL	108.1 (677.5)	37.0 (187.5)	305.5 (1462.8)	<0.001
Ferritin, ug/L	1529.0 (1496.5)	1224.5 (2426.0)	1744.0 (2422.3)	0.094
D-dimers, ug/mL	3.3 (5.3)	1.2 (2.9)	1.7 (3.0)	0.138
CRP, mg/L	88.0 (149.6)	32.5 (124.6)	111.0 (203.5)	0.001
LDH, U/L	575.0 (436.5)	487.0 (192.8)	515.5 (311.0)	0.695
Procalcitonin, ng/mL	0.3 (1.8)	0.2 (0.5)	0.8 (3.3)	0.059
Fibrinogen, g/L	4.9 (4.1)	3.5 (3.4)	4.7 (3.8)	0.386
ESR, mm/h	40.0 (66.0)	27.5 (45.0)	45.0 (75.0)	0.066
Leucocytes, ×10^3^/uL	12.7 (9.3)	17.0 (9.5)	11.9 (7.8)	<0.001
% Lymphocytes	5.2 (5.6)	3.7 (2.9)	7.4 (8.6)	0.117
Lymphocytes, ×10^3^/uL	0.6 (0.5)	0.7 (0.4)	0.7 (1.0)	0.639
BUN, mg/dL	54.0 (38.5)	71.5 (55.8)	56.2 (61.1)	0.036
Creatinine, mg/dL	0.8 (0.4)	0.9 (0.6)	0.7 (1.0)	0.208
pH	7.4 (0.2)	7.4 (0.1)	7.4 (0.1)	0.974
Lactate, mmol/L	2.3 (1.1)	2.3 (1.2)	2.2 (1.2)	0.891

Data reported as *n* (%) and calculated using Chi-square test and Fisher’s exact unless specified differently; IQR—interquartile range; IL-6—interleukin 6; CRP—C-reactive protein; LDH—lactate dehydrogenase; ESR—erythrocyte sedimentation rate; BUN—blood urea nitrogen.

**Table 3:** Laboratory findings of patients with severe COVID-19 after TPE.

Variables—Median (IQR)	1 TPE (*n* = 41)	2 TPE (*n* = 13)	>2 TPE (*n* = 11)	*p*-Value	*p*-Value *
IL-6, pg/mL	76.0 (371.6)	17.9 (142.6)	156.0 (320.5)	<0.001	<0.001
Ferritin, ug/L	1141.0 (1469.5)	722.5 (962.5)	1307.0 (2105.5)	0.073	0.126
D-dimers, ug/mL	1.5 (3.8)	1.5 (3.2)	1.8 (3.1)	0.618	0.070
CRP, mg/L	65.0 (86.5)	20.0 (38.1)	98.2 (150.5)	<0.001	0.119
LDH, U/L	417.0 (248.0)	395.5 (227.8)	424.5 (281.3)	0.695	0.042
Procalcitonin, ng/mL	0.2 (1.7)	0.2 (0.4)	0.8 (3.4)	0.086	0.388
Fibrinogen, g/L	3.4 (1.8)	2.4 (2.1)	3.3 (2.5)	0.426	0.097
ESR, mm/h	15.0 (22.5)	11.0 (10.0)	20.0 (41.3)	0.044	<0.001
Leucocytes, ×10^3^/uL	15.2 (12.5)	18.5 (11.1)	14.2 (8.8)	0.121	0.003
% Lymphocytes	5.6 (8.2)	4.3 (5.6)	6.2 (9.5)	0.204	0.088
Lymphocytes, ×10^3^/uL	0.7 (0.6)	0.8 (0.7)	0.8 (1.1)	0.630	0.517
BUN, mg/dL	51.6 (48.3)	65.6 (51.6)	64.0 (54.9)	0.219	0.365
Creatinine, mg/dL	0.8 (0.6)	0.8 (0.6)	0.8 (1.0)	0.658	0.551
pH	7.4 (0.1)	7.4 (0.1)	7.4 (0.1)	0.906	0.925
Lactate, mmol/L	2.3 (1.1)	2.3 (1.2)	2.3 (1.3)	0.833	0.749

Data reported as *n* (%) and calculated using Chi-square test and Fisher’s exact unless specified differently; IQR—interquartile range; IL-6—interleukin 6; CRP—C-reactive protein; LDH—lactate dehydrogenase; ESR—erythrocyte sedimentation rate; BUN—blood urea nitrogen; * significance measured between the values before TPE and one day after TPE.

**Table 4:** Outcomes of patients with severe COVID-19 by number of TPE sessions.

Variables	1 TPE (*n* = 41)	2 TPE (*n* = 13)	>2 TPE (*n* = 11)	*p*-Value
MAP before, mmHg (mean ± SD)	80.3 ± 13.0	83.8 ± 14.6	79.9 ± 11.8	0.768
MAP after, mmHg (mean ± SD)	75.0 ± 17.0	88.7 ± 12.6	80.6 ± 11.2	0.023
Body temperature before, Celsius (mean ± SD)	36.5 ± 0.4	36.4 ± 0.4	36.6 ± 0.7	0.573
Body temperature after, Celsius (mean ± SD)	36.5 ± 0.5	36.6 ± 0.4	36.7 ± 0.7	0.364
SOFA score before TPE	7.6 ± 3.9	7.0 ± 2.7	8.2 ± 4.0	0.733
SOFA score after TPE	7.9 ± 3.8	6.7 ± 3.0	8.3 ± 3.9	0.507
APACHE 2 score before TPE	12.4 ± 4.9	11.2 ± 5.0	11.5 ± 4.2	0.685
APACHE 2 score after TPE	13.3 ± 5.6	11.2 ± 5.4	11.7 ± 4.1	0.391
Oxygenation index before TPE	24.1 ± 12.2	21.2 ± 5.6	20.0 ± 10.1	0.456
Oxygenation index after TPE	22.5 ± 13.1	22.8 ± 8.1	19.7 ± 10.2	0.763
ROX index before TPE	6.5 ± 4.6	7.4 ± 3.0	11.4 ± 4.8	0.007
ROX index after TPE	5.9 ± 5.2	8.2 ± 3.2	13.1 ± 5.4	<0.001
HACOR score before TPE	6.2 ± 0.4	6.0 ± 1.1	6.0 ± 0.1	0.415
HACOR score after TPE	7.0 ± 2.2	5.5 ± 1.1	5.8 ± 0.4	0.019
PaO2/FiO2 before TPE	106.6 ± 58.7	133.3 ± 65.8	144.3 ± 85.9	0.161
PaO2/FiO2 after TPE	109.3 ± 52.9	137.4 ± 57.2	146.7 ± 83.8	0.110
PaO2/FiO2 > 100mmHg before TPE (*n*,%)	13 (34.1%)	8 (61.5%)	5 (45.5%)	0.208
PaO2/FiO2 > 100mmHg after TPE (*n*,%)	11 (26.8%)	7 (53.8%)	3 (27.3%)	0.178
Intubated, (*n*,%)	18 (43.9%)	5 (38.5%)	5 (45.5%)	0.927
5-day mortality	20 (48.8%)	5 (38.5%)	3 (27.3%)	0.411
Overall mortality, (*n*,%)	32 (78.0%)	9 (69.2%)	6 (54.5%)	0.290

Data reported as *n* (%) and calculated using Chi-square test and Fisher’s exact unless specified differently; IQR—interquartile range; MAP—mean arterial pressure; SOFA—sequential organ failure assessment; APACHE—acute physiology and chronic health evaluation; PaO2/FiO2—pressure of arterial oxygen to fractional inspired oxygen concentration ratio; P/F—pressure of arterial oxygen to fractional inspired oxygen concentration.

## Data Availability

Data available on request.
